# Distinct Characteristics of COVID-19 Infection in Children

**DOI:** 10.3389/fped.2021.619738

**Published:** 2021-03-04

**Authors:** Xuejiao Han, Xuemei Li, Yinan Xiao, Ruoning Yang, Yang Wang, Xiawei Wei

**Affiliations:** ^1^Laboratory of Aging Research and Cancer Drug Target, State Key Laboratory of Biotherapy and Cancer Center, National Clinical Research Center for Geriatrics, West China Hospital, Sichuan University, Chengdu, China; ^2^Quality Management Department, Southwestern Hospital, Army Medical University, Chongqing, China; ^3^West China School of Medicine, West China Hospital, Sichuan University, Chengdu, China

**Keywords:** pediatrics, COVID-19, SARS-C0V-2, infection, characteristics, children

## Abstract

SARS-CoV-2, a member of the family coronaviridae, has triggered a lethal pandemic termed coronavirus disease 2019 (COVID-19). Pediatric patients, mainly from families with a cluster of infection or a history of exposure to epidemic areas, get infected via direct contacts or air-borne droplets. Children (aged below 18 years) are susceptible to COVID-19, with an average incubation period of about 6.5 days. Most cases present asymptomatic or common cold symptoms such as fever, cough, and myalgia or fatigue, which is milder than adult patients. Besides, most abnormal laboratory and radiologic findings in children with COVID-19 are non-specific. Since no specific chemotherapeutic agents have been approved for children, timely preventive methods could effectively forestall the transmission of SARS-CoV-2. To date, mostly studied cases have been adults with COVID-19, whereas data on pediatrics patients remain poorly defined. We herein conducted a literature review for papers published in PubMed and medRxiv (preprints) between December 2019 and December 2020 that reported on pediatrics patients (aged below 18 years) with a confirmed COVID-19 diagnosis. In this review, we summarized and discussed the pathogenesis, epidemiology, and clinical management of COVID-19 in pediatrics patients to improve our understanding of this new disease in children.

## Introduction

In December 2019, a new type of pneumonia of unknown etiology quickly spread. An unknown beta-CoVs was detected by unbiased sequencing in the samples from patients with new pneumonia (1), which was later termed SARS-CoV-2 by the International Committee on Taxonomy of Viruses (ICTV) based on its close relationship with SARS-CoV. Both SARS-CoV and MERS-CoV belong to beta-CoVs and share 79.6 and 50% identity with SARS-CoV-2 (2, 3). Currently, COVID-19 has spread widely around the world, affecting more than 200 countries and territories. The population of all ages is susceptible to COVID-19 via respiratory tract infection or direct contact due to the absence of specific immunity (4).

Even though the overall mortality for children is about 1% and severe complications are less likely to occur, the possibility of children being infected is the same as that of adults (5). The difference in disease development between children and adults may lead to distinct clinical management. Therefore, we conducted a literature review for papers published in PubMed and medRxiv (preprints) between December 2019 and December 2020 that reported on pediatrics patients (aged below 18 years) with a confirmed COVID-19 diagnosis. In this review, we focus on the pathogenesis, epidemiologic features, clinical symptoms, diagnostic criteria, and prevention methods of children with COVID-19, and expect to provide systematic understanding and new insight into the diagnosis as well as management of children infected with COVID-19.

## Pathogenesis

SARS-CoV-2 enters cells by binding to the receptor, angiotensin-converting enzyme 2 (ACE2) through S protein (6). ACE2 is highly expressed in alveolar epithelium, heart, renal tubules and intestinal epithelial cells (7–9). Severe complications of COVID-19, such as multiple organ dysfunction syndromes (MODS) and acute respiratory distress syndrome (ARDS), might be caused by dysregulated immune response and cytokine storms (10–12). Recently, many severe cases presenting persistent fever and the involvement of two or more organ systems in children with COVID-19 have been reported, which is termed as multisystem inflammatory syndrome (MIS-C). The symptoms of MIS-C are similar to Kawasaki Disease (KD) and toxic shock-like syndrome (13). MIS-C was firstly reported in Europe (13, 14). Pediatrics patients diagnosed with MIS-C are less common in Asia countries than in Europe, with only one case in Korea now (15). Some studies hypothesized that the pathogenesis of MIS-C might be attributable to genomic variation of virus and post-infectious immune dysregulation (16). Recently, immunophenotype studies have demonstrated that some MIC-S patients have a peculiar B cell response with an increase of plasmablast (PB) proportion (17, 18). However, the specific pathological process is still unclear, and further studies evaluating the etiopathogenesis of MIS-C are essential for developing the treatment strategies.

## Epidemiologic Features

Children of all ages are susceptible to COVID-19 (19). Despite the higher incidence of COVID-19 in older children, infants (<1 year) seem to be the most vulnerable due to a high hospitalization rate (20). The average incubation period for COVID-19 in children is about 6.5 days, which is longer than the 5.4 days reported in adults (21). There is no significant gender difference in pediatric patients (19). Of the 2,135 pediatric patients, 94.1% of cases were either asymptomatic or diagnosed with a mild to moderate symptom, only 5.8% had developed severe complications compared with 18.5% in adult patients (19). Several factors could account for severe outcomes among children with COVID-19 infection, such as immunocompromised condition, pulmonary pathology, the age of infants (<3 months) and patients' underlying diseases, including asthma and obesity (22).

Even though the symptoms of pediatric patients are mild, children are as susceptible as adults. Recent virologic data indicated that viral load in the asymptomatic patient was not significantly different from that in the symptomatic patients, which suggested asymptomatic patients could be the potential source of COVID-19 infection (23, 24). In addition, nasopharyngeal SARS-CoV-2 viral loads in infected children were similar to those in other age groups, indicating that children were at a similar risk of infection to adults (25). However, all data suggest that SARS-CoV-2 transmission from children to adults or other children is infrequent (26). The incidence rate of COVID-19, though low in pediatric patients, varies from study to study in different countries (Table 1). The variation of incidence rate is probably due to the testing policy and testing availability. According to the report from the Centers for Disease Control and Prevention (CDC) in the United States, SARS-CoV-2 pediatrics patients (<18 years) have accounted for 10.2% of all reported cases by December 14, 2020 (39). However, mortality in infected children is <1% (39).

**Table 1:** Clinical characteristics among COVID-19 pediatrics patients in different countries (27–38).

**Characteristics**						
**Region**	**USA**	**China**	**Italy**	**Spain**	**Iran**	**Mexico**
Incidence (%)	1.7	12.3	NA	16.6	NA	1.5
Number of patients	2,572	171	100	58	30	51
Median age (range), yr	11 (0–17)	6.7 (1 day−15 yr)	3.3 (0–17.5)	2.9 (3.3 months−12.2 yr)	5.5 (1 day−15 yr)	10
**Age distribution, No. (%)**						
<1 yr	398 (15.5)	31 (18.1)	40 (40.0)	NA	6 (20.0)	8 (15.7)
1 to <6 yr	NA	40 (23.4)	15 (15.0)	NA	11 (36.7)	8 (15.7)
6–10 yr	NA	58 (33.9)	21 (21.0)	NA	NA	NA
>10 yr	NA	42 (24.6)	24 (24.0)	NA	NA	NA
Male-No./total No. (%)	1,408/2,490 (56.5)	104/171 (60.8)	57/100 (57.0)	37/58 (63.8)	14/30 (46.7)	26/51 (51.0)
Coexisting conditions-No./total No. (%)	80/345 (23.2)	NA	27/100 (27.0)	23/58 (39.7)	7/30 (23.3)	8/51 (15.7)
**Exposure to SARS-COV-2-No./total No. (%)**						
Family cluster	168/184 (91.3)	131/171 (76.6)	45/100 (45.0)	30/58 (51.7)	NA	NA
Other exposure	16/184 (8.7)	2/171 (1.2)	48/100 (48.0)	NA	NA	NA
Unknown exposure	0	15/171 (8.8)	7/100 (7.0)	NA	19/30 (63.3)	18/51 (35.3)
Survived-No./total No. (%)	2,569/2,572 (99.9)	170/171 (99.4)	100/100 (100.0)	57/58 (98.3)	30/30 (100.0)	48/51 (94.1)
**Symptoms-No./total No. (%)**						
Fever	163/291 (56.0)	71/171 (41.5)	54/100 (54.0)	41/58 (70.7)	23/30 (76.7)	40/51 (78.4)
**Temperature**						
≤37.5°C	128/291 (44.0)	100/171 (58.5)	46/100 (46.0)	NA	NA	NA
37.6-38.0°C	NA	16/171 (9.4)	15/100 (15.0)	NA	NA	NA
38.1–39.0°C	NA	39/171 (22.8)	28/100 (28.0)	NA	NA	NA
>39.0°C	NA	16/171 (9.4)	11/100 (11.0)	NA	NA	NA
Cough	158/291 (54.3)	83/171 (48.5)	44/100 (44.0)	42/58 (72.4)	16/30 (53.3)	34/51 (66.7)
Diarrhea	37/291 (12.7)	15/171 (8.8)	9/100 (9.0)	7/58 (12.1)	3/30 (10.0)	7/51 (13.7)
Rhinorrhea	21/291 (7.2)	13/171 (7.6)	22/100 (22.0)	33/58 (56.9)	0	10/50 (20.0)
Fatigue	NA	13/171 (7.6)	9/100 (9.0)	NA	NA	NA
Shortness of breath	39/291 (13.4)	NA	11/100 (11.0)	10/58 (17.2)	NA	NA
Sore throat	71/291 (24.4)	NA	4/100 (4.0)	4/58 (6.9)	6/30 (20.0)	10/49 (20.4)
Nausea/Vomiting	31/291 (10.6)	NA	10/100 (10.0)	9/58 (15.5)	8/30 (26.7)	5/49 (10.2)
Abdominal pain	17/291 (5.8)	NA	4/100 (4.0)	NA	NA	NA
Headache	81/291 (27.8)	NA	4/100 (4.0)	8/58 (13.8)	NA	28/49 (57.1)
Myalgia	66/291 (22.7)	NA	NA	2/58 (3.4)	NA	NA
Runny nose	21/291 (7.2)	NA	NA	NA	0	NA
**Region**	**Argentina**	**Brazil**	**Ethiopia**	**Perú**	**UK**	**Korea**
Incidence (%)	NA	19.1	NA	19.9	0.8	NA
Number of patients	578	66	90	91	451	91
Median age (range), yr	4.2 (0.7–11.2)	7.0 (24 day−18 yr)	15 (6 months−18 yr)	6 (3–10)	3.9 (0.3–12.9)	11 (0.07–18)
**Age distribution, No. (%)**						
<1 yr	NA	13 (19.7)	NA	NA	162 (35.9)	6 (6.6)
1 to <6 yr	NA	NA	NA	NA	NA	13 (14.3)
6–10 yr	NA	NA	16 (17.8)	NA	NA	23 (25.3)
>10 yr	NA	NA	64 (71.1)	NA	150 (33.2)	49 (53.8)
Male-No./total No. (%)	315/578 (54.5)	44/66 (66.7)	33/90 (36.7)	58/91 (63.7)	256/450 (56.9)	53/91 (58.2)
				49/91 (53.8)		
Coexisting conditions-No./total No. (%)	204/578 (35.3)	50/66 (75.8)	3/90 (3.3)		195/451 (43.2)	6/91 (6.6)
**Exposure to SARS-COV-2-No./total No. (%)**						
Family cluster	NA	NA	NA	24/91 (26.4)	NA	57/91 (62.6)
Other exposure	NA	NA	NA	4/91 (4.4)	NA	30/91 (33.0)
Unknown exposure	156/578 (27.0)	39/66 (59.0)	49/90 (54.4)	63/91 (69.2)	NA	4/91 (4.4)
Survived-No./total No. (%)	577/578 (99.8)	65/66 (98.5)	90/90 (100.0)	82/91 (90.1)	448/451 (99.3)	91/91 (100.0)
**Symptoms-No./total No. (%)**						
Fever	207/400 (51.7)	37/66 (56.1)	5/90 (5.6)	36/91 (39.6)	306/418 (73.2)	62/91 (68.1)
Temperature						
≤37.5°C	NA	NA	NA	NA	NA	NA
37.6−38.0°C	NA	NA	NA	NA	NA	35/91 (38.5)
38.1–39.0°C	NA	NA	NA	NA	NA	NA
>39.0°C	NA	NA	NA	NA	NA	NA
Cough	40/400 (10.0)	23/66 (34.8)	20/90 (22.2)	18/91 (19.8)	175/431 (40.6)	37/90 (41.1)
Diarrhea	NA	NA	NA	NA	58/431 (13.4)	11/90 (12.2)
Rhinorrhea	39/400 (9.7)	NA	NA	NA	NA	24/90 (26.7)
Fatigue	NA	NA	5/90 (5.6)	NA	103/431 (23.9)	5/89 (5.6)
Shortness of breath	NA	10/66 (15.2)	NA	12/91 (13.2)	124/389 (31.9)	1/77 (1.3)
Sore throat	49/400 (12.2)	NA	9/90 (10.0)	NA	40/431 (9.3)	22/77 (28.6)
Nausea/Vomiting	NA	NA	4/90 (4.4)	11/91 (12.1)	120/380 (31.6)	6/90 (6.7)
Abdominal pain	15/400 (3.7)	NA	NA	NA	66/431 (15.3)	6/77 (7.8)
Headache	37/400 (9.2)	NA	9/90 (10.0)	NA	43/431 (10.0)	12/77 (15.6)
Myalgia	NA	NA	NA	NA	28/431 (6.5)	7/77 (9.1)
Runny nose	NA	NA	5/90 (5.6)	NA	61/431 (14.1)	NA

Different from the various transmission routes of adults, pediatric patients get infected mainly from families with a cluster of infection or a history of exposure to epidemic areas (40). On January 11, 2020, in Shenzhen, Guangdong, China, one asymptomatic child (aged 10 years) was confirmed, whose parents and grandparents suffered COVID-19 earlier (4). Although schools and universities have been fully reopened, few infected cases were identified, suggesting that children play a potentially minor role in SARS-CoV-2 transmission within schools and beyond (41). On the other hand, Kang Zhang et al. reported that real-time polymerase chain reaction (RT-PCR) results in rectal swabs were persistently positive even after nasopharyngeal swabs turned negative (42). Therefore, they hypothesized the fecal-oral transmission and doubted whether children could facilitate it if they were not toilet trained. ACE2 is highly expressed in stratified epithelial cells of upper esophageal and intestinal epithelial cells in the ileum and colon (9). And the fecal-oral transmission does exist with other respiratory viruses (43). Although fecal-oral transmission has not been confirmed, it cannot be ruled out.

The reason for the lower infection rate among children might be the closure of schools and kindergartens reduces the exposure of children to the virus, and children are not tested for SARS-CoV-2 as frequently as adults due to mild or absent symptoms. For example, in a study of children (aged below 22 years) tested for SARS-CoV-2 at a community, the result showed that 28.2% had a positive PCR test, much higher than the reported incidence of COVID-19 in pediatric patients (44).

## Clinical Presentation

The clinical manifestations of pediatric patients are mostly mild and non-specific. Common symptoms among these early confirmed patients included fever, cough, and myalgia or fatigue (45–47). The median duration of fever in children lasts 3 days compared to 10 days in adults (48). A few children have upper respiratory symptoms, such as nasal congestion, sore throat and runny nose (4, 46). Specifically, gastrointestinal symptoms could be initial symptoms in some cases, including nausea, vomiting, diarrhea and abdominal pain, and these pediatric patients are more likely to develop the more severe clinical condition (49, 50). And gastrointestinal symptoms are twice as common in children as in adults (51, 52). Some infected newborns may present only low spirits, loss of appetite, and shortness of breath (22, 53). Severe pediatric cases show dyspnea and cyanosis and may advance to ARDS, septic shock, refractory metabolic acidosis, MODS, and coagulation dysfunction (21, 50). According to one analysis from China, younger children, especially infants and pre-school children are more susceptible to severe symptoms (10.6% <1-year-old vs. 3%≥16 years old) (19). The potential explanation is the immaturity of the immune system in infants and pre-school children. Recently, Kawasaki-like disease was reported among children with COVID-19 (13). This disease is also named pediatric inflammatory multisystem syndrome (PIMS) or MIS-C due to its clinical manifestation associated with multisystem inflammation such as conjunctivitis, myocarditis, meningitis and coronary vessel inflammation (13, 54). Feldstein et al. reported that 33% of the diagnosed infected children had Kawasaki-like clinical symptoms (55). Intriguingly, a 14-year-old boy diagnosed and treated for orchiepididymitis, however, confirmed COVID-19 infection without respiratory symptoms, suggesting the possibility of testicular involvement in COVID-19 (56). Different clinical features of pediatrics patients among countries are summarized in Table 1.

**Figure 1:**
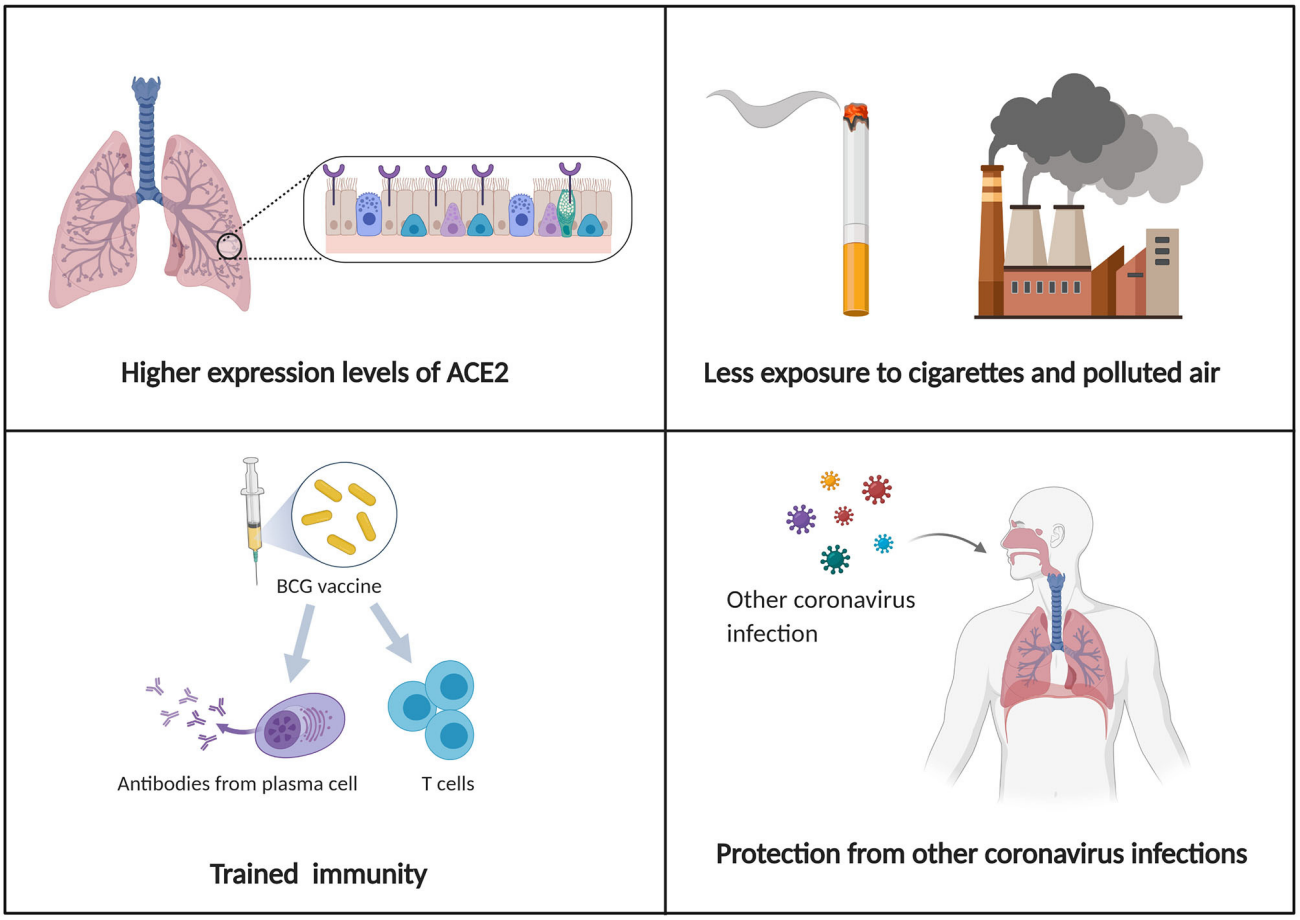
Schematic illustration of possible mechanisms for explaining why children have milder symptoms and a better prognosis than adults.

The reason why children have milder symptoms than adults remains unclear. According to recent research, several factors are worth considering (Figure 1). The first reason is the higher expression level of ACE2 in children. According to the analysis from China, older adults (>50 years) who were more likely to develop into serious pneumonia, presented decreased expression of ACE2 when compared to children. This may impact disease severity and recovery from pneumonia caused by SARS-CoV-2 infection in older patients (57). The second possibility is trained immunity, which means training innate immunity to generate immune memory for non-specific immune protection (58). Thirdly, the difference in innate and adaptive immunity between adults and children should also be taken into account. And the antibodies following other coronavirus infections may play a protective role in SARS-CoV-2 infection (59). Finally, compared with the elderly, children had no underlying diseases but a healthy respiratory tract without exposure to cigarettes and polluted air.

## Laboratory and Radiologic Examinations

Most abnormal laboratory findings in children with COVID-19 are non-specific. The white blood cell count is typically normal or reduced with decreased lymphocyte and/or neutrophil counts (27, 60). The levels of C-reactive protein and procalcitonin (PCT) can be normal or elevated. However, children have a much lower prevalence of increased C-reactive protein than adults, suggesting a much milder immunological response and less immune damage (61). The best markers for diagnosing the severity of the disease in children are the levels of bilirubin and hepatic enzymes (62, 63). During April 2020, a surge of PIMS cases presenting a hyper-inflammatory state including elevated levels of C-reactive protein, PCT, ferritin, and D-dimers, as well as markers of myocarditis (13).

RT-PCR is the most common to detect SARS-CoV-2 nucleic acid. Since the virus exists in serum, urine, stool, upper, and lower respiratory tract specimens, nasopharyngeal and oropharyngeal swabs, bronchoalveolar lavage, or tracheal aspirates might be helpful (4, 64). The result from Liu et al. showed that the median viral shedding duration detected in nasopharyngeal swabs, oropharyngeal swabs, and stools were 13, 4, and 43 days, respectively, suggesting the possibility of fecal-oral transmission (65). Neutralizing antibody (NAb) response is also helpful for the diagnosis. However, negative antibody tests cannot exclude COVID-19 as it needs a certain time period for the body to produce serum-specific antibodies after infection (65).

The sensitivity of chest X-ray might be lower than that of computed tomography (CT) scan. Infected pediatric patients do not commonly present abnormalities in chest X-ray at the early stage, with occasional interstitial changes (66). Chest CT abnormalities observed in pediatric patients include unilateral or bilateral multiple patchy shadows, nodular ground-glass opacities (GGO) or consolidations with a surrounding halo sign (67). The sensitivity of chest CT in diagnosing COVID-19 is greater than that of RT-PCR (98 vs. 71%) (68). In addition, lung ultrasound (LUS) is also used as a diagnostic tool. Recent research from Italy indicated LUS abnormalities including subpleural consolidations and confluent B-lines in eight children with COVID-19 infection (69).

## Diagnosis

The criteria for COVID-19 diagnosis in children are based on epidemiology, clinical manifestations, and laboratory testing to confirm SARS-CoV-2 infection. The case definition and clinical classification of children are summarized in Table 2. Also, co-infections such as mycoplasma, influenza A and B, respiratory syncytial virus, Epstein-Barr virus, cytomegalovirus, parainfluenza, and adenovirus should take into consideration in diagnosis since co-infections rate are up to 79% in children (67).

**Table 2:** Case definition and clinical classification of children from the Chinese updated consensus statement (March 24, 2020) (22).

**CASE DEFINITION**
A suspected case is defined as a case that meets: one epidemiologic criterion and two clinical criteria
**Epidemiologic criteria:** 1. Children with a travel or residence history in a community with infected cases reported in China or a country or region with a serious epidemic within 14 days prior to disease onset (with the global pandemic of COVID-19, imported cases deserve attention) 2. Children with a history of contacting patients infected with SARS-CoV-2 within 14 days prior to disease onset 3. Children with a history of contacting patients with fever or respiratory symptoms from communities with reported cases in China or countries or regions with serious epidemic within 14 days prior to disease onset 4. Clustered cases: two or more cases with fever and/or respiratory symptoms within 14 days in small groups (such as family members, school classmates, etc.) 5. Newborns delivered by mothers with confirmed infection.
**Clinical criteria:** 1. Fever, fatigue, dry cough, and/or other respiratory symptoms; some pediatric patients may have low-grade fever or no fever 2. Patients with the following chest imaging findings: single or multiple localized ground-glass opacities in the form of light cloud or fine mesh, with thickened blood vessels shadows inside the lesions; localized consolidation, located under the pleura or near the bronchial blood vessel bundles, most in the bilateral lower lobes of the periphery of the subpleural lung; increased ground-glass shadows; large-scale consolidation; diffused consolidation of unilateral or bilateral lungs, with ground-glass opacities, bronchial inflation signs 3. In the early phase of the disease, white blood cell count is normal or decreased, or with decreased lymphocytes count 4. No other pathogens are detected which can fully explain the clinical manifestations.
**A confirmed case is defined as a case that meets any of the following criteria:** 1. Testing positive for SARS-CoV-2 by real-time PCR 2. Genetic sequencing of respiratory tract or blood samples is highly homologous with the known SARS-CoV-2 3. Both serum-specific antibodies IgM and IgG are positive 4. Serum-specific antibody IgG changed from negative to positive or increased 4-folds or higher than that in the acute phase during the recovery period.
**CLINICAL CLASSIFICATION**
1. Asymptomatic infection (silent infection) Testing positive for SARS-CoV-2, but without clinical symptoms or abnormal chest imaging findings 2. Acute upper respiratory tract infection With only fever, cough, pharyngeal pain, nasal congestion, fatigue, headache, myalgia or discomfort, etc., and without signs of pneumonia by chest imaging or sepsis 3. Mild pneumonia With or without fever, with respiratory symptoms such as cough; and chest imaging indicating changes of viral pneumonia, but not reaching the criteria of severe pneumonia 4. Severe pneumonia a. Polypnea: ≥60 times/min (<2 months), ≥50 times/ min (2–12 months), ≥40 times/min (1–5 years), ≥30 times/min (>5 years) (after ruling out the effects of fever and crying) b. Oxygen saturation <92% under a resting state c. Dyspnea: assisted breathing (moans, nasal flaring, and three concave sign), cyanosis, intermittent apnea d. Disturbance of consciousness: somnolence, coma, or convulsion e. Food refusal or feeding difficulty, with signs of dehydration f. Pulmonary high-resolution CT (HRCT) examination showing bilateral or multi-lobe infiltrates, rapid progression of disease in a short period or with pleural effusion. 5. Critical cases (require ICU care) a. Respiratory failure requiring mechanical ventilation b. Shock c. Combined with other organs failure.

## Therapy and Prevention

Until now, no concrete evidence has demonstrated the effectiveness and safety of specific drugs against COVID-19. Children with mild or absent symptoms should be isolated at home for 2 weeks. Severe cases should be admitted to the pediatric intensive care unit (PICU) as soon as possible. Antiviral drugs targeting specific sites on different stages could effectively inhibit the virus replication in the host cells. However, their efficacy and safety remain to be determined. Antibiotics and antifungal drugs can be used only in patients with secondary bacterial infections based on the culture and antibiogram results. Given the immunomodulatory effect, corticosteroids are critical for the treatment of inflammatory and immune diseases. However, the modulatory property sometimes might lead to immune suppression that hinders the virus clearance in the host. Therefore, corticosteroids should be avoided, except when required for other indications such as MIS-C, refractory shock, or asthma exacerbation. Evidence showed that the adjunctive steroid treatment was effective and safe in pediatric patients with Kawasaki-like presentations. However, they showed resistance to intravenous immunoglobulin (IVIG) (13). In November 2020, the US Food and Drug Administration (FDA) provided Emergency Use Authorizations (EUA) for two novel virus-neutralizing monoclonal antibodies (mAbs) for the treatment of mild to moderate COVID-19 in adolescents and adults in specified high-risk groups. However, the safety and efficacy of mAbs for the COVID-19 treatment among children or adolescents remains unclear (70). Most pediatric patients with COVID-19 present a good prognosis and usually recover within 1–2 weeks. Despite a higher hospitalization rate of infants, children are rarely admitted to intensive care units (ICU) in general (71).

Emergent health conditions all over the world accelerate vaccine development, clinical testing, and usage. The vaccine currently available mainly falls into six categories: inactivated virus, live attenuated virus, nucleic acid-based vaccines, protein subunit vaccines, virus-like particles (VLPs), and recombinant viral vectors (72). Until January 21, 2021, 64 vaccine candidates have been under clinical development while 10 vaccines (inactivated vaccines, RNA-based vaccines, and non-replicating viral vectors) have been adopted for “emergent use” in America, Canada, China, Russia, Brazil, etc. (73, 74).

Variability of host immune responses among populations, production of secreted IgA antibodies on the mucosal surface in the upper respiratory tract, and T-cell involvement in the immune response all play roles in eliciting successful protection against SARS-CoV-2 (75). An increasing number of clinical trials have demonstrated the safety and immunogenicity of SARS-CoV-2 vaccines among people ranging from 18 to 55 years old, with a low incidence of side effects such as fatigue and headache (76–78). However, lacking data concerning the safety and response rate in licensed clinical trials, vaccine efficacy in children remains unclear. It is noteworthy that Pfizer recently has included children aged from 12 to 17 into clinical trials of mRNA vaccine, which might feedback the assessment of its effectiveness shortly (79). Additionally, the occurrence of rare severe side effects such as vaccine-associated enhanced disease and Pediatric Inflammatory Multisystem Syndrome Temporally associated with SARS-CoV-2 (PIMS-TS) suggests that researchers should further explore the different immunopathogenesis of COVID-19 between children and adults (80, 81). Vaccine-associated enhanced disease refers to severe side effects related to worse clinical outcomes after vaccination vs. without vaccination (80). In the 1960s, due to atypical measles such as fever and pneumonia observed in children after formalin-inactivated measles virus vaccination, the vaccine was prohibited (82). Despite the absence of compelling evidence regarding VAED in both animal models and human beings (80), additional attention to such rare adverse immune responses could guarantee the overall safety of populations administrated vaccines. Finally, reinfection interval or the longest possible immunity is also worth noticing since it helps indicate the optimal age for vaccination, thus providing better protective strategies for children against COVID-19 (83).

Since no specific drugs against COVID-19, it is crucial to prevent the amplification of the outbreak. Early detection of children, a possible hidden source could effectively prevent the outbreak in kindergartens or schools, especially for children who tend to develop atypical clinical characteristics but with a high virus load. Once confirmed with COVID-19 infection, the children must be quarantined at home or in the hospital to prevent close contact and human-to-human transmission. Social distance and washing hands frequently are crucial to prevent COVID-19 spread. In addition, consistent use of the mask is necessary, except for children under age two or anyone who has trouble removing it on his own. Besides, children should maintain good moods, exercise regularly and have a balanced diet to enhance their immunity (22).

## Conclusions

This review summarized the updated evidence regarding the epidemiology and clinical management of COVID-19 in children. Even though most pediatric patients with COVID-19 present mild symptoms and good prognosis, children are as susceptible as adults. Besides, an increasing number of COVID-19 pediatric patients with MIS-C have been reported. Further study is of paramount importance for better prevention, diagnosis and treatment of COVID-19 in children worldwide.

## Author Contributions

XH and XL were major writers of the manuscript. YX and RY designed the tables and edited the manuscript. YW researched appropriate references and reviewed the manuscript. XW developed the structure of the article, reviewed and edited the manuscript. All authors read and approved the final manuscript.

## Conflict of Interest

The authors declare that the research was conducted in the absence of any commercial or financial relationships that could be construed as a potential conflict of interest.
